# Effects of human activity on the habitat utilization of Himalayan marmot (*Marmota himalayana*) in Zoige wetland

**DOI:** 10.1002/ece3.7733

**Published:** 2021-06-07

**Authors:** Shuailing Zhou, Ali Krzton, Shuai Gao, Cheng Guo, Zuofu Xiang

**Affiliations:** ^1^ College of Life Science and Technology Central South University of Forestry & Technology Changsha China; ^2^ Department of Research and Instruction RBD Library Auburn University Auburn AL USA

**Keywords:** burrow feature, burrow site selection, habitat utilization, Himalayan marmot, human activities

## Abstract

Human activity is increasingly and persistently disturbing nature and wild animals. Affected wildlife adopts multiple strategies to deal with different human influences. To explore the effect of human activity on habitat utilization of Himalayan marmot (*Marmota himalayana*), habitat utilization patterns of three neighboring marmot populations in habitats affected differently by human activities were recorded and compared. We found that (a) distance between reproductive burrows (a represent of reproductive pairs) becomes shorter under the influence of human activities, and more burrows were dug as temporary shelters, resulting in shorter distance between those shelters and shorter distance flee to those shelters and, consequently, shorter flight initiation distance when threatened. More burrows that are closer to the disturbed habitats improve the ability to escape from threats. (b) Reproductive burrow site selection of the species is determined by the availability of mounds in the habitat, and breeding pairs selectively build reproductive (also the hibernation) burrows on mounds, potentially to improve surveillance when basking and the drainage of burrows. Human activities generally drive breeding pairs away from the road to dig their reproductive burrows likely to reduce disturbance from vehicles. However, even heavy human activity exerts no pressure on the distance of reproductive burrows from the road or the mound volume of the high disturbance population, potentially because mounds are the best burrowing site to reproduce and hibernate in the habitat. Marmots deal with disturbance by digging more burrows in the habitat to flee more effectively and building reproductive burrows on mounds to gain better vigilance and drainage efficiency.

## Graphical abstract

To explore the effect of human activity on habitat utilization of the Himalayan marmot (*Marmota himalayana*), habitat utilization patterns of three neighboring marmot populations in habitats affected differently by human activities were recorded and compared. Marmots residents in Zoige wetland deal with nonlethal human disturbance by digging more burrows in the habitat to flee more effectively and building reproductive burrows on mounds to gain better vigilance and drainage efficiency.

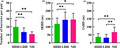

## 1 INTRODUCTION

Nature is increasingly affected by human disturbances around the world. With the human population growing, more than 80% of global land surfaces are affected by human activities (Sanderson et al., 2002). Besides affecting environments on a macro level, human activities also affect aspects of wildlife interaction with those environments such as distribution, population dynamics, and ability to survive in changing conditions (Gül et al., 2018; Trombulak & Frissell, 2000; UNEP, 2001).

Human activities generally exert direct and indirect negative effects on animals. Direct and fatal disturbances include both illegal poaching and legal hunting (Brockman et al., 2020; Ménard et al., 2014), road killing by vehicles (Richini‐Pereira et al., 2008), which will kill victims directly, and sometimes result in a population decline of some species (Rija et al., 2020), and damage regional community structure (Clark et al., 2015; Trombulak & Frissell, 2000). Indirect and less fatal effects include habitat degradation, traffic noise, light pollution, or hunting‐derived competition between different species, which will trigger reduced reproductive output and decline in body condition of affected animals (French et al., 2011; Hellgren & Polnaszek, 2011; Muhly et al., 2011; Primack, 2008; Safina & Burger, 1983; Webber et al., 2013) and may result in local extinction at population level due to habitat removal (Griffin et al., 2007; Imperio et al., 2013). Furthermore, species that accompany humans, such as domestic dogs (*Canis lupus familiaris*), also negatively impact the survival of wild animals (Mainini et al., 1993; Mori, 2017). On the other hand, some animals benefit from human activity. For instance, some prey species experience reduced mortality because humans drive their predators and/or competitors away from human‐dominated habitats (Hebblewhite et al., 2005; Lambe, 2016; Muhly et al., 2011). Some species have improved feeding efficiency due to human activities (Marty et al., 2019; Xiang et al., 2011) or gain higher reproductive success due to better nesting conditions in areas with human activity (O'Donnell & Denicola, 2006), benefits that can directly promote the population growth.

Different animals have greater or lesser chances to survive in the face of different human disturbances (Amphibiaweb, 2021; Imperio et al., 2013; Lambe, 2016; Ménard et al., 2014) depending upon the type and degree of human activities (Griffin et al., 2007; Ménard et al., 2014), as well as the species’ ability to adapt to disturbance (Griffin et al., 2007; Muhly et al., 2011; Webber et al., 2013; Yang et al., 2019). Possible outcomes for these populations include either coexistence with humans or active avoidance of humans (Braczkowski et al., 2018; Griffin et al., 2007; Magle et al., 2005), or local extinction (Amphibiaweb, 2021; Imperio et al., 2013). Generally, small‐bodied species may survive more easily in areas of intense human activity than bigger species and even benefit from the altered landscape. For example, red foxes (*Vulpes vulpes*) occur at higher densities in the city than in rural areas because of the absence of coyotes (*Canis latrans*), and some urban‐living macaques (*Macaca* spp.) obtain better food relative to their rural populations (Lambe, 2016; Marty et al., 2019). On the other hand, large‐bodied species tend to avoid habitats impacted by humans regardless of whether humans actively kill them (Klaassen & Broekhuis, 2018; Macedo et al., 2018; Paudel & Kindlmann, 2012). Though in rare cases, populations forced to share habitats with humans, such as leopards (*Panthera pardus*) in Mumbai, India, develop particular strategies like adjusting their daily time budget and prey selection to survive (Braczkowski et al., 2018). Additionally, some animal species adopted different strategies and have different destinies under different human disturbances, depending on the type and intensity of disturbances (Austin & Ramp, 2019; Jahren et al., 2020; Murdoch et al., 2016).

Especially, highly residential species with limited migration ability and low phenotypic plasticity are at the greatest risk of going locally extinct due to human disturbance whether they are big‐ or small‐bodied. For example, the Yunnan lake newt (*Cynops wolterstorffi*; Amphibiaweb, 2021), Alpine rock ptarmigans (Imperio et al., 2013), and Asiatic lion (*Panthera leo persica*) as well as south China tiger (*P. tigris amoyensis*) who cannot avoid human disturbances in the form of roads or log through migration (Jhala et al., 2019; Tilson et al., 2004), the population decline and local extinctions are common. Nevertheless, some certain other residential species like some rodents (Harris & Munshi‐South, 2017; Maher, 2009), primates (Marty et al., 2019), and some carnivores like some red fox populations (Jahren et al., 2020; Lambe, 2016) are better able to adapt and survive in human‐dominated habitats and gain a higher population density relative to their rural congeners. To deal with different human influences suffered, animals have adopted multiple survival strategies such as adjusting time rhythm (Poudel et al., 2015a), allocating more time to vigilance (Griffin et al., 2007; Poudel et al., 2015b), or using habitats farther away from human activity (Macedo et al., 2018; Paudel & Kindlmann, 2012; Pita et al., 2020). In terms of the effect of human activities on habitat utilization for animals that can survive disturbances that are not directly fatal, certain strategies were adopted to deal with different disturbances. For example, Vancouver Island marmot (*Marmota vancouverensis*) may build additional burrows for shelter when threatened (Blumstein et al., 2001), bamboo rat (*Rhizomys sinensis*) selectively construct their burrows away from roads (Yuan et al., 2017), or some grassland species like alpine marmot (*M. marmota*) select regions with large stones to allow better vigilance (Borgo, 2003). Furthermore, species like alpine marmot and some waterbirds can behaviorally reduce flight initiation distance (FID) to optimize their fitness by the accustomed to nonfatal human activities (Feng & Liang, 2020; Louis & Le Berre, 2000; Thibault et al., 2020).

Marmots (*Marmota* spp.) are large, residential ground‐dwelling, and burrowing squirrels with relatively weak ability to disperse and high philopatry (Armitage et al., 2011; Griffin et al., 2007), forcing them to continue exploiting habitats disturbed by humans (Neuhaus & Mainini, 1998). Previous studies illustrated that Himalayan marmots (*M*. *himalayana*) deal with grazing disturbances by adjusting their daily time rhythm (Poudel et al., 2015a) and changing the time allocated to feeding and vigilance behavior (Poudel et al., 2015b). In comparison, some other marmot species like yellow‐bellied marmots (*M. flaviventris*) and Olympic marmots (*M. olympus*) also adjust the time spent on feeding and vigilance, and further, they also adjust their FID when disturbed by different human activities (Griffin et al., 2007; Li et al., 2011). On the contrary, the FID of woodchuck (*M. monax*) did not vary along a rural–urban gradient, but the home range of the species decreased with the increasing urbanization (Watson, 2009). Besides, the study on alpine marmots indicated that they have learned to tolerate hikers that pass by Mainini et al. (1993).

Himalayan marmots are mainly distributed across the Qinghai–Tibetan Plateau (Shrestha, 2016). Some regional populations suffer persistent disturbance from human activities such as extermination campaigns to prevent disease, which subsequently has caused them to increase their reproductive rate in the years following these population reductions (Huang et al., 1986; Wang et al., 1986). Other populations are indirectly disturbed by domesticated yaks and goats, resulting in changes to time spent feeding and greater feeding efficiency (Poudel et al., 2015a, 2015b). The effects of persistent, but not fatal, human disturbances on the Himalayan marmot require further investigation. For example, the impact of motor vehicle activity on their habitat utilization, population dynamics, and behavioral plasticity is still underexplored (Edwards et al., 2019; Kitchen et al., 1999; Klaassen & Broekhuis, 2018; Whittington et al., 2019). In the present study, we recorded and compared the patterns of habitat utilization of three Himalayan marmot populations sharing the same habitat type, but suffering different levels of anthropogenic disturbance around a Tibetan village in the Zoige wetland (Guo et al., 2020), to explore the effects of human activity on this species’ behavior and discover changes that might improve their survival. Because reproductive pairs of the marmot will dig some temporary burrows as a shelter when threatened (Blumstein et al., 2001) and human did not alter their habitat selection in the region (Guo et al., 2020), we predict that (a) the distance between burrows of each breeding pair will decrease with increasing human activity as a consequence of population growth; (b) more temporary burrow will be dug, and consequently, the distance between burrows will become shorter with increasing human activity; (c) as a consequence of more refuge and reduced interburrow distance, the FID of disturbed populations will become shorter relative to unaffected population; (d) the distance from reproductive burrows to the nearest road will become longer with increasing human activity; and (e) due to the absence of large rocks in the region, marmots impacted by human disturbance will preferentially build reproductive burrows on sites that allow for better surveillance of the area, such as big mounds occurring on the grasslands.

## 2 MATERIALS AND METHODS

### 2.1 Study site and animals

This study was conducted around Duoma (103.01°E, 33.5°N), a village approximately 8.5 km southwest of the town of Ruoergai County in the Zoige wetland, the biggest plateau peat bog in the world (Zhang et al., 2005). The Zoige wetland is located in the eastern Qinghai–Tibet Plateau, southwestern China. The study site is a mosaic of grasslands, ground frost heaves, rivers, and wet and dry wetland patches (Guo et al., 2020), and according to some local elders, the village has been here for at least 80 years.

**FIGURE 1:**
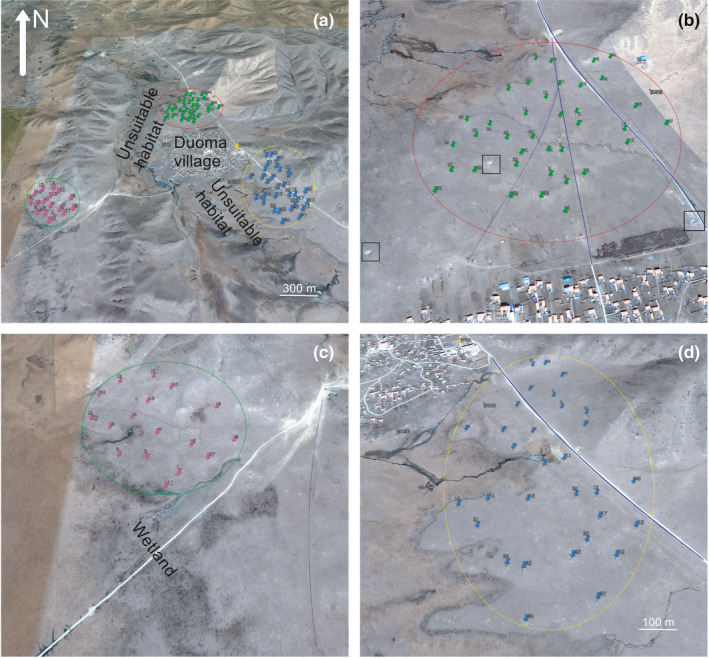
Map of the study site and location of the three study populations. Blue lines represent roads. (a) The region circled in red represents HDH, the region circled in yellow represents LDH, and the region circled in green represents minimally disturbed NH. (b) Area of the HDH. Black rectangles represent garbage dumps. Green pushpins represent the location of reproductive burrows. (c) Area of the habitat with minimal disturbance (NH). Pink pushpins represent the location of reproductive burrows. The white line in the figure is a makeshift road in the wetland and is generally abandoned by residents, and they prefer the road marked with a blue line. (d) Area of the LDH. Blue pushpins represent the location of reproductive burrows

The three marmot populations have been the subject of an ongoing behavioral ecology study since 2017; they live in different locations around the village and share the same habitat type (dry, flat patches with short grass and few frost heaves in the wetland; Guo et al., 2020; Figure S1), but suffer different degrees of anthropogenic disturbances. The marmots living in front of the village, hereafter the high disturbance habitat (HDH), are persistently disturbed by the daily activities of local residents including passing motor vehicles and stray dogs. This interference does not directly kill marmots and does not alter their preferred habitat type (i.e., expel them from selected habitat to other unsuitable habitats; Guo et al., 2020; Figure S1). Marmots living behind the village in the low disturbance habitat (LDH) endure relatively fewer disturbances than those living in front of the village. A third population living to the west of the village lives in a minimally disturbed natural habitat (NH) and serves as a control group (Figure 1). We had no direct interactions like a routine collection of blood or tissue samples or simulated behavioral experiments with them during the burrow‐related data collection in 2019. To further examine the burrow diversification‐derived flee strategy under different human disturbances, some individual‐based FID data were collected in June 2020 as an additional experiment to explore how human activities act on their habitat utilization features.

### 2.2 Sampling method and statistical analyses

During the marmots’ active period (not in hibernation) in 2019, we classified the intensity of human disturbance of each habitat based on the degree of pressure from human activity on the different groups recorded during behavioral observations in 2018. The three study groups were designated as living in the HDH, the LDH, and the NH. To quantify the amount of human activity in the area, we recorded how many automobiles, motorcycles, and stray dogs passed by the marmot habitat every 15 days from April 20 to October 5, 2019.

In each of the three habitats, we recorded the coordinates of reproductive burrows (the most extensively used burrows) and temporary burrows (used only occasionally for shelter) of every breeding pair; the pair‐specific burrows were determined according to behavioral observation. The natural feature where all burrows occurred (i.e., hummock/mound or flat ground) and the physical parameters (long diameter, short diameter, and height) of the mounds were also recorded to calculate their volume where burrows are located (simplify mound into a cone). The locations of all burrows were mapped in Google Earth to find (a) the distance between each (adjacent) reproductive burrow, and reproductive burrows with geographical connectivity only were included in the following analysis. For example, the distance between NH8 and NH5, LDH3, and LDH14 was excluded in the subsequent analysis because they are isolated by a ditch (Figures S4 and S5). A criterion finally results in 51, 47, and 31 inter‐reproductive burrow distances for HDH, LDH, and NH; (b) the distance between all burrows (distance between temporary burrows, temporary burrows, and reproductive burrows and 50 distances were randomly selected in each habitat to conduct the subsequent analysis); and (c) the distance from some reproductive burrows to the nearest road (only burrows next to the road with no other reproductive burrows between them and the road like HDH17, HDH18, and LDH1 were included; for detail, please see Figures S2 to S5 and sheet named “distance to road” in Table S1). We calculated the density of breeding pairs by linking the outermost burrows recorded to form a perimeter and measured number of pairs inside and link the outermost burrows of each breeding pair to calculate pair‐specific home‐range size. The same procedure was applied to all three populations. Specially for NH, because there is no direct disturbance from motor vehicles in the site, the same as the criterion in two disturbed habitats, the distance from the outermost reproductive burrow (NH2; Figure S5) to the nearest road was used as the standard distance (311 m) to the road for all burrows in the NH.

One adult individual in several pairs from each habitat was randomly selected to measure the FID. A field assistant held binoculars from a long distance to observe and record, while Zhou Shuailing approached the focal marmot at a speed of 1 m/s until the marmot started to run. FID (the distance between Zhou and the start point of the flee) of the focal individual was then measured (Blumstein et al., 2004). Finally, 28 FID samples from HDH, 20 from LDH, and 20 from NH (three more individuals from other undisturbed pairs were also included in the analysis) were included in the following analysis.

A chi‐square test was used to determine (a) whether there was seasonal variation in different human activities, (b) diversification of breeding pair density between each habitat, and (c) the variation of reproductive den site location in three habitats. A *t* test was used to determine (a) whether the intensity of different human activities was significantly different among the three habitats, (b) whether differences in parameters such as the number of burrows per reproductive pair, the distance between reproductive burrows and between all burrows, and the distance between reproductive burrows and the corresponding nearest road were significantly different by population, and (c) the diversification of mound measurement (volume) selected as reproductive burrow site among different habitats. Besides, a *t* test was also used to test whether there was diversification on the FID of individuals and pair‐specific home‐range size in different populations. All statistics were conducted in SPSS 20.0.

## 3 RESULTS

### 3.1 Differences in intensity of human disturbance

**FIGURE 2:**
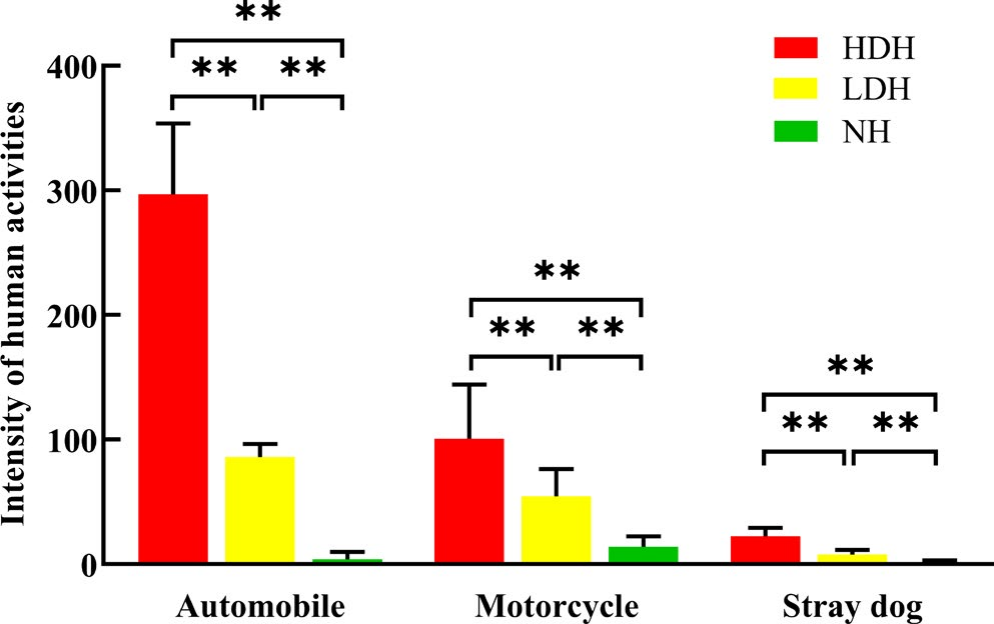
Statistics on the intensity of different human activities within each habitat across the observation period (the number of automobiles, motorcycles, and stray dogs was periodically counted and then compared in *t* test directly to illustrate the diversification of intensity). *p < .05, **p < .01

The intensity of different human activities differed significantly among the three study habitats: the mean number of automobiles every observation day passing through the HDH (297.00 ± 56.7) is significantly more than LDH (86.08 ± 10.44) and NH (4.00 ± 5.96; Figure 2); besides, both motorcycles (100.83 ± 43.4 vs. 54.58 ± 21.67 vs. 14.00 8.43) and stray dogs (22.4 ± 7.2 vs. 7.75 ± 3.77 vs. 1.00 ± 2.00) showed the same trend too (Figure 2). Both HDP and LDP suffered relatively intensity, persistent and evenly influences from automobile, motorcycle and dog during the whole active period of the species, (i.e. about 300 and 86 automobiles per observation day passing through the two disturbed habitats; Figure 2; Table 1). However, in NH, except for a dozen motorcycles passing by every observation day, there is seasonal fluctuation in the frequency and number of automobile and stray dog incursion into the habitat (i.e., about 12 automobiles per observation day from the end of June to early August only) due to the routine pasture rotation of local residents (Table 1).

**TABLE 1:** Seasonal variation in the intensity of human activities (measured in the number of daily different human activities) in high disturbed habitat (HDH), low disturbed habitat (LDH), and NH measured according to chi‐test

	Automobile	Motorcycle	Stray dog
HDH	0.83	0.83	1.33
LDH	0.83	0	2.00
NH	11.33**	1.33	16.00**

*p* values of all significant results are less than 0.01.

** *p* < .01.

### 3.2 Differences in habitat utilization

**FIGURE 3:**
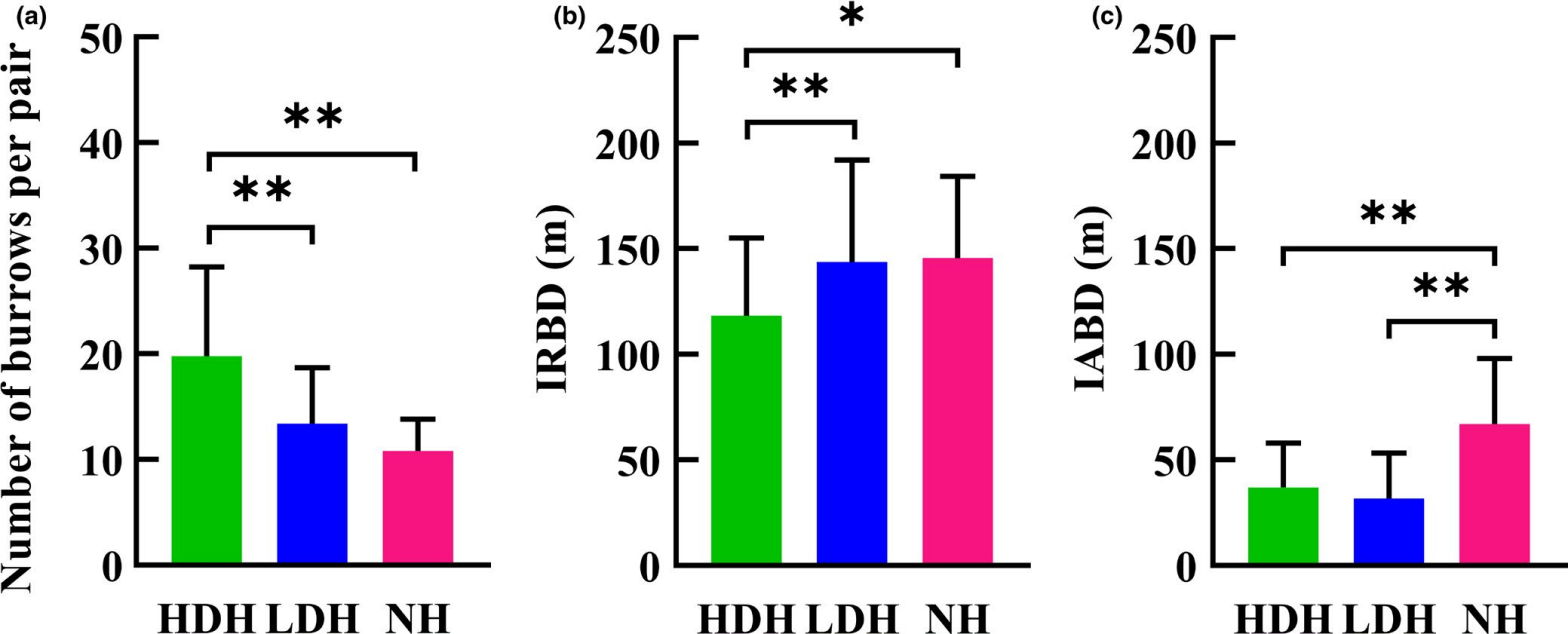
Statistics on (a) number of burrows per breeding pair in the three habitats, (b) distance between adjacent reproductive burrows (IRBD), and (c) distance between all burrows (IABD) in the three habitat conditions. *p < .05, **p < .01

The density of breeding pairs was 72 pairs per km^2^ in the HDH, 50 pairs per km^2^ in the LDH, and 55 pairs per km^2^ in the NH (Table 2; Figure 1b–d), although none of the differences in breeding pair density were detected among the three habitats are statistically significant (χ^2^ = 1.14, *p* = .285 between HDH and NH; χ^2^ = 1.99, *p* = .157 between HDH and LDH; χ^2^ = 0.119, *p* = .729 between LDH and NH). However, intergroup differences emerged in measurements related to the burrows themselves, with a significant negative correlation between the intensity of human activity and the number of burrows per breeding pair: pairs in HDH dig far more (twice as much as) pair‐specific burrows than pairs in LDH (*t* = 3.63, *p* = .000, *df* = 66) and NH (*t* = 4.21, *p* = .000, *df* = 52); furthermore, though not significant (*t* = 1.83, *p* = .074, *df* = 46), pairs in LDH also on average dig two more extra burrows than their counterparts in NH (13.39 ± 0.96 vs. 10.82 ± 0.73; Figure 3a).

**TABLE 2:** The number and density of breeding pair and burrows in high disturbed habitat (HDH), low disturbed habitat (LDH), and NH

	Area (km^2^)	Number of breeding pair^a^	Pair density (/km^2^)^b^	Number of all burrow	Burrow density (/km^2^)
HDH	0.51	37	72	694	1,361
LDH	0.62	31	50	350	565
NH	0.31	17	55	167	539

^a^ The same as number of breeding burrow.

^b^ The same as density of breeding burrow.

**FIGURE 4:**
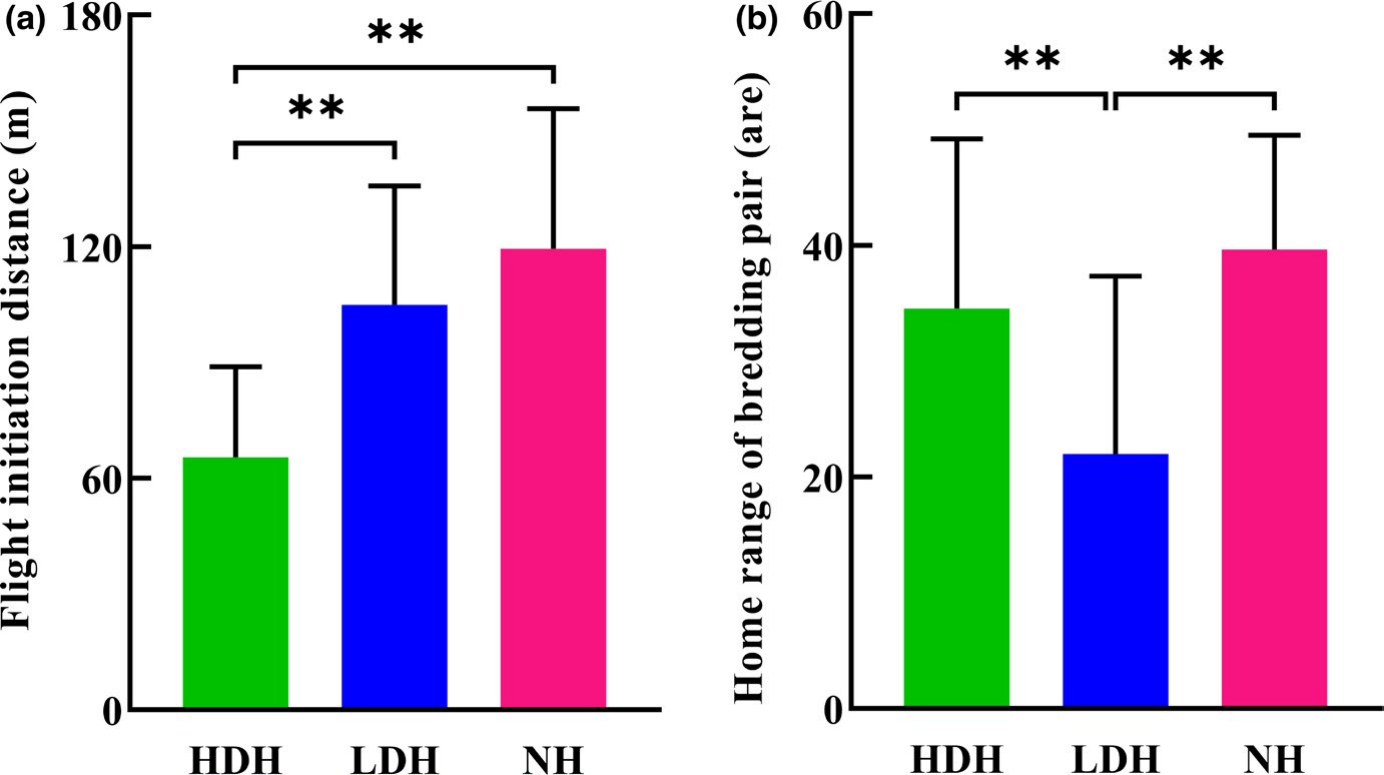
Statistics on flight initiation distance of individuals (a) and home‐range size (b) of breeding pairs in three habitats. *p < .05, **p < .01

Moreover, average distance between burrows also differs between different habitats: Interburrow distance of reproductive burrows in the HDH is less than that of the other two habitats (*t* = −3.22, *p* = .002, *df* = 81 relative to NH and *t* = −2.95, *p* = .004, *df* = 96 relative to LDH), although no significant difference was found between the low disturbance and NHs (*t* = −0.18, *p* = .86, *df* = 77; Figure 3b). As for the distances between all burrows in the habitat, relative to the NH, human activities led to the same decline in the interburrow distance in two disturbed habitats (Figures 2 and 3c). Besides, the FID of individuals in HDP derived from inter‐all‐burrow distance is shortest among all three habitats as expected (Figure 4a); nevertheless, though inter‐all‐burrow distances in LDP are the shortest among three habitats (Figure 3c), and the home‐range size of pairs in LDH is significantly smaller than pairs in other two habitats (*t* = −3.34, *p* = .001, *df* = 62 relative to HDH, and *t* = −4.02, *p* = .000, *df* = 42 relative to NH; Figure 4b), FID of individuals in the LDP are longer than marmots in HDP (*t* = 5.05, *p* = .000, *df* = 46) and no differentiation emerged relative to individuals from NP (*t* = 1.36, *p* = .182, *df* = 38; Figure 4a).

**FIGURE 5:**
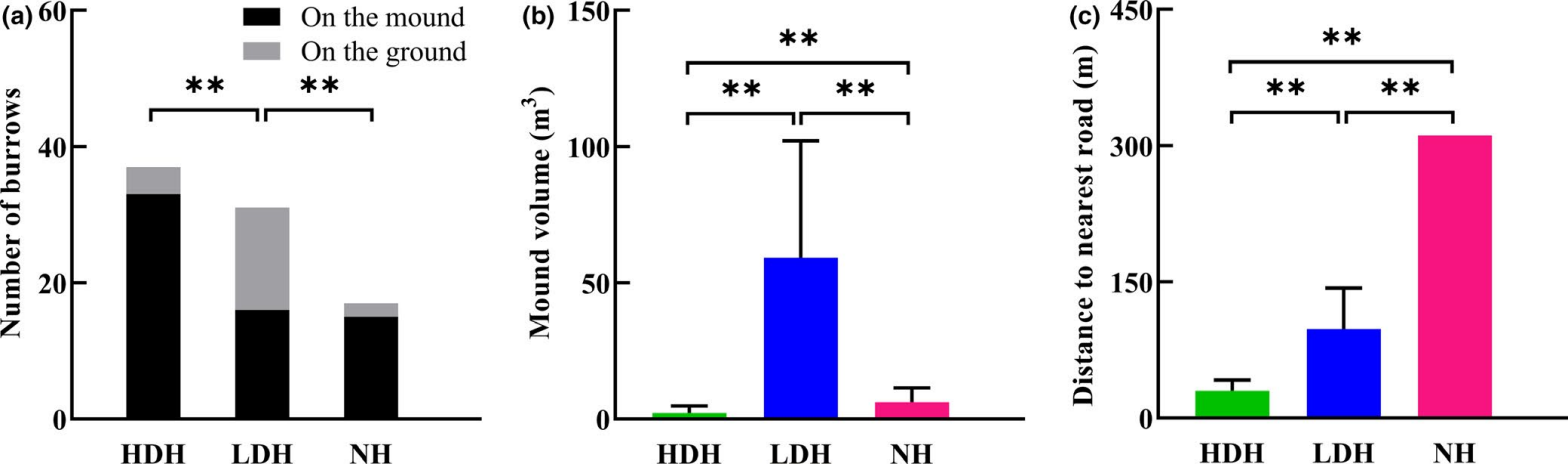
Burrow site selection of breeding pairs in the three habitats. (a) The location and number of reproductive burrows by habitat. (b) The volume of mounds selected as reproductive burrow sites by habitat. (c) The distance of reproductive burrow to the nearest road by habitat. *p < .05, **p < .01

Similarly, the characteristics of sites selected for the digging of reproductive burrows also differed depending on human activity levels. Relative to pairs in the low disturbance population, both reproductive pairs in the high disturbance population (χ^2^ = 7.28, *p* = .007) and the natural population (χ^2^ = 5.89, *p* = .015) preferentially constructed their reproductive burrows on mounds raised above the level of the surrounding ground (Figure 5a). The volume of those mounds also differed between sites, with mounds used for reproductive burrows in the high disturbance population being significantly smaller than mounds in the NH (*t* = −2.68, *p* = .014, *df* = 19.7), and both of those habitats’ mounds being much smaller than the mounds selected by pairs in the low disturbance population (Figure 5b). Finally, the mean distance from reproductive burrows to the nearest road in the HDH is significantly shorter than in the LDH (*t* = −5.77, *p* = .000, *df* = 15.97; Figure 5c).

## 4 DISCUSSION

We found that as a residential species that have to share the same space with human, pairs of Himalayan marmots in Zoige wetland tended to dig more burrows relative to pairs in NH, resulting in shorter interburrow distances if they are suffered persistent but nonfatal disturbance from human activities (Bryant, 1996; Griffin et al., 2007) due to their high phenotypic plasticity (Huang et al., 1986; Poudel et al., 2015a, 2015b). Besides, most breeding pairs in the region generally prefer to construct their reproductive burrows on mounds. Specifically, relative to pairs in HDH selectively dig their reproductive burrows on mounds whether the mound volume or the distance to road, pairs in the LDH did not show the same preference but on average dug their reproductive burrows away from roads.

Different from the fatal threatens like poaching and habitat loss that will directly kill affected animals (Rija et al., 2020; Tilson et al., 2004), residents in our study site never kill marmots due to their religious faith, but their daily activities are a persistent disturbance for the animals, and the level of disturbance differs between the habitats due to different road locations and the number of motor vehicles passing through. Stray dogs are a deadly threat to marmots (Poudel et al., 2015a), but although they are more abundant in the HDH and LDH, several garbage dumps in the area are capable of supplying enough food for them (Figure 1b), and dogs around the village do not go out of their way to hunt marmots within their range (Altmann & Muruthi, 1988). No stray dog predation on marmots was observed during our fieldwork, the main human influence on the marmot populations comes from motor vehicles that pass through the habitat. Seasonal fluctuation in the intensity of automobiles and dogs in the NH occurs due to residents driving by with their dogs during the annual seasonal rotation of pasture, while daily trips between the village and pastures are done by motorcycles (Table 1).

All marmot breeding pairs dig a reproductive burrow for regular use to rest, reproduce, and hibernate, but they also dig temporary burrows, which are occupied less frequently, throughout the home range as a refuge when threatened (Blumstein et al., 2001; Zhang et al., 2019). All breeding pairs around the village dig multiple burrows for shelter; nevertheless, pairs in HDH dug more pair‐specific burrows (19.76 ± 1.4) than pairs in the LDH (13.4 ± 0.96) and NH (10.8 ± 0.73), probably due to they suffer the heaviest disturbance (Figure 2). Though nonsignificant, pairs in LDH generally dig two more burrows than NH pairs, more available refuges guarantee individuals have more chances of escape and consequently provide a survival advantage when threatened (Blumstein et al., 2001). Furthermore, shorter inter‐all‐burrow distances resulting from more burrows in the habitats enable marmots in two disturbed habitats to reach a potential refuge more quickly when threatened, increasing the likelihood of survival (Li et al., 2011; Zaman et al., 2019). Based on observations recorded from 2017 to 2020, no new temporary burrows were dug. It is possible that more burrows were dug in the HDH during the initial human settlement of the area, but marmots that had grown accustomed to humans’ daily activities no longer saw a benefit to digging new burrows (Mainini et al., 1993; Schell et al., 2018), which is energetically expensive.

Similarly, though only two more temporary burrows were dug, inter‐all‐burrow distance in LDH is far shorter than in NH, allowing the same reduction in distance and time required to reach a safe place for individuals in the habitat as their congeners in HDH. The different (number of burrows per pair) and the same (inter‐all‐burrow distance) patterns that emerge between two disturbed habitats may arise because the disturbances LDP individuals suffer are not intense enough to accustom them, but drive they selectively concentrate new burrows near reproductive burrows like urban woodchucks (Watson, 2009), the mean home range of LDP pairs (21.98 ± 2.86 are) is far smaller than pairs in NH (39.62 ± 2.55 are) with there are many unoccupied regions among different pairs in LDP (Figure S4), consequently, gain shorter inter‐all‐burrow distance to meet the requirements of flee efficiency and spend as little energy as possible on digging extra burrows simultaneously. Meanwhile, FID of HDP individuals (65.36 ± 4.45 m) are shorter than NP individuals (119.40 ± 8.11 m) as expected, nevertheless, even have the shortest inter‐all‐burrow distance, the FID of LDP individuals (105.00 ± 6.88 m) showed no coincident trend as HDH, but are as long as FID of marmots in NP (Dill & Houtman, 1989; Griffin et al., 2007). The differentiation may arise because the optimal strategy to survival for LDP individuals is to flee early like NP individuals when threatened however the distance to a potential refuge (Li et al., 2011). Shorter flee distance and longer FID guarantees the safety of unaccustomed LDP individuals under the disturbances of human activities (Feng & Liang, 2020; Zaman et al., 2019).

It is also worth noting that the inter‐reproductive burrow distance in HDH (118.31 ± 36.82 m) is shorter than that of the other two habitats (Figure 3b); a pattern may arise because the regions surrounding the HDH are uninhabitable due to improper soil and vegetation characteristics (Guo et al., 2020; Figure S1). HDP is actually an isolated population that cannot freely communicate with other populations. The same as a reintroduced alpine marmot population in Dolomiti Bellunesi National Park, Italy (Borgo et al., 2009), the HDH has been fully exploited by the growing breeding pairs since the village began to settle in the region. 72 pairs per km^2^ may be the maximum environmental carrying capacity for the species in such an ecosystem. In contrast, no similar variation emerged between LDP and NP; this may be because LDH is an open area conducive to free dispersal as the NH (Figures S2 to S5). This might explain why the interburrow distance for reproductive burrows in the LDH was no different than that observed in the NH. LDH is an open space, marmots in the region have the freedom to actively avoid human influences in emigration, a strategy that is superior to the passive adaptation to human influence. The average inter‐reproductive burrow distances observed in LDH (143.73 ± 48.25 m) and NH (145.57 ± 38.66 m) may reflect more typical distancing between marmot pairs, reducing resource competition while maintaining regular contact between pairs. Together with the diversification on FID and two interburrow distances, we concluded that compared with HDP, the reactions of LDP individuals may be the normal outcomes (dig more extra and concentrated temporary burrows and flee earlier to avoid potential dangers but also appropriate inter “family” distance) when Himalayan marmot affected by persistent, but nonfatal disturbances from humans.

The characteristics of reproductive den site selection also differed among the habitats. Most NH pairs constructed their reproductive burrows on mounds, and pairs in HDH also selectively dig their reproductive burrows on mounds (Figure 5a), even when those mounds were relatively close to a road and smaller than the mounds used by pairs in NH (Figure 5b,c). Marmots use their reproductive burrows giving birth to their offspring and spending a lot of time resting/basking at the entrance to the burrow. This special preference to mound may be because pairs build their reproductive burrows on mounds ensure better drainage relative to burrows dug on flat ground (Szor et al., 2007). Besides, similar to alpine marmots preferentially remaining near large stones that they climb to engage in surveillance to watch for predators more effectively (Borgo, 2003), Himalayan marmots in alpine meadow with less mound also selectively use site with many big stone to gain better vigilance and bask efficiency (Figure S6). However, in our site in Zoige wetland, due to the lack of large stone, rest or vigilance on mound higher than flat ground may also be able to gain a better vision of the surrounding areas, improving their chances of detecting predators.

**FIGURE 6:**
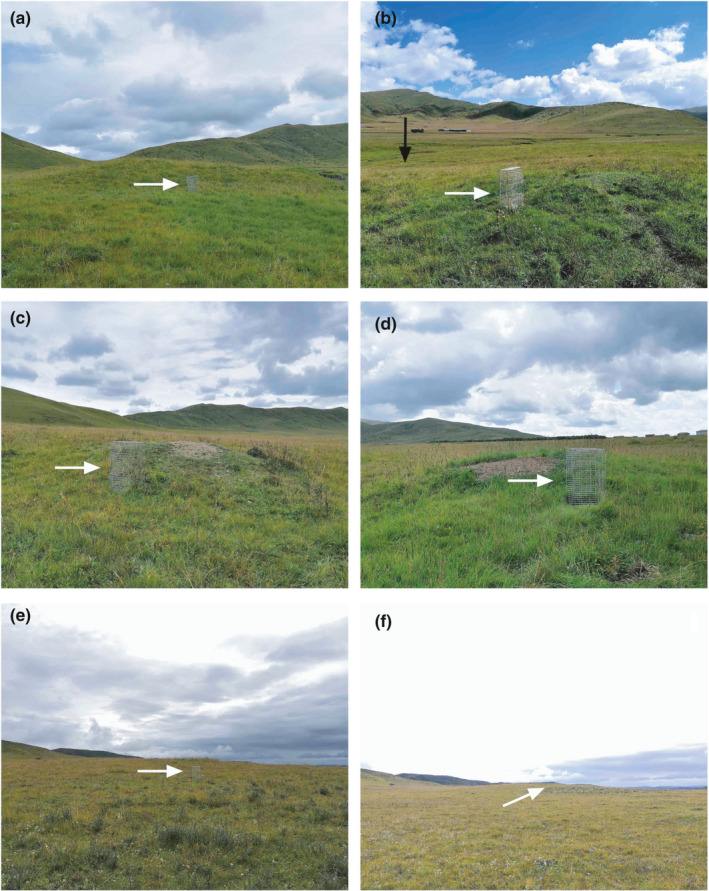
The shape and size of mound selected as den site in different habitats. (a) Natural mound in NH. (b) Natural mound in high disturbed habitat. (c) Constructed mound in NH. (d) Constructed mound in high disturbed habitat. (e, f) Hill in low disturbed habitat. The white arrows in the pictures illustrate the cage (80 cm in height) as the reference and the black arrow in figure B is the road next to a reproductive burrow (HDH 13: Figure S2)

Most animals choose to locate reproductive dens at sites where they can conceal themselves to better protect themselves and their offspring (Lai et al., 2020; May et al., 2012; Ross et al., 2010; Sazatornil et al., 2016). Consequently, we predicted that pairs of Himalayan marmot would stay as far from the roads as possible, but breeding pairs in HDH still preferentially built their reproductive burrows on the mounds near roads despite the increased frequency of disturbance from the motor vehicles, which can be harmful (Whittington et al., 2019). This surprising result suggests that the availability of mounds is the primary determinants of site selection for reproductive burrows in Himalayan marmots. In Zoige wetland, mounds on the dry flat ground could be the limiting resource (Guo et al., 2020), as marmots always built burrows in the mounds that were present regardless of their size or distance from the road. For example, one occupied mound (HDH11) in HDH was only 2.2 m from a road (Figure S3), and the average size of the occupied mounds in the HDH is smaller (2.14 ± 2.65 m^3^) than the occupied mounds in the NH (6.23 ± 5.13 m^3^; Figure 6a,b), indicating that marmots will use all the mounds they can find in an area, even smaller ones. There were no unoccupied mounds left in the HDH, and some breeding pairs that could not find a natural mound will built their own very small mounds around the entrance of their burrows (Figure 6c,d). There are no natural hiding places for marmots in the Zoige wetland (Zhang, 2019), and unlike predators, disturbances from daily human activities are nonfatal, and consequently, sites that allowed for vigilance while resting were the only suitable choices for reproductive burrows, even if they were frequently disturbed by motor vehicles. Den site selection of American black bears (*Ursus americanus*) and American badger (*Taxidea taxus*) and the habitat utilization of Barbary macaques (*Macaca sylvanus*) were also found to be unaffected by the distance to roads (Sunga et al., 2017; Waller et al., 2013; Waterman et al., 2019), suggesting that many species will tolerate persistent but non‐life‐threatening human disturbance to retain access to otherwise favorable habitat. The importance to the marmots of the vigilance and good drainage of mound‐built burrows (Szor et al., 2007) outweighed disadvantages to digging reproductive burrows close to a road. Furthermore, dig their reproductive burrows near road may also arise because relative to other species sensitive to human disturbance (i.e., snowy plover *Charadrius nivosus* and Yunnan lake newt; Amphibiaweb, 2021; Webber et al., 2013), marmots species are more able to endure nonfatal human disturbances (Griffin et al., 2007; Neuhaus & Mainini, 1998). Himalayan marmots disturbed in HDH for generations are highly accustomed to human activities, consequently, disturbances from different human activities are no longer selective pressures on the den site selection of individuals in the habitat. (Schell et al., 2018).

Site selection for reproductive burrows in the LDH showed a different profile relative to the other two populations, with burrows almost equally likely to be located on mounds or on flat ground. Moreover, the average volume of the mounds selected for reproductive burrows in the LDH is significantly larger (75.47 ± 78.69 m^3^) than the mounds in HDH and NH. This discrepancy might result from the radically different topography of the area. Aside from having many large mounds, the LDH is sloped, with some areas of the flat ground allowing for surveillance equal to the tops of mounds in the other two habitats (Figure 6e,f). Consequently, pairs in LDH are no longer limited by the availability of mounds. This is consistent with the greater average distances from reproductive burrows to the road in the LDH (98.06 ± 48.06 m) as opposed to the HDH (28.88 ± 12.29 m). Unlike the marmots of HDP, who are forced to prioritize vigilance and drainage, marmots in LDH have greater flexibility in sites where they can build reproductive burrows and so tend to avoid the roads.

Unlike reproductive burrows, temporary burrows were common on flat ground in all three habitats because they were used only to evade immediate threats. Good vision and drainage are not important for temporary burrows (Borgo, 2003; Szor et al., 2007). Consequently, Himalayan marmots dig temporary burrows in any location as needed and reserve their reproductive burrows for mounds when possible. This demonstrates the use of multiple habitat utilization strategies at once to cope with human disturbance and natural dangers.

Generally, relative to animals sensitive to human activities like Yunnan lake newt or some certain populations suffer extensive human disturbance like Asiatic lions, Himalayan marmot have a high plasticity, variation in habitat utilization in response to the varied intensity of nonfatal human disturbance of the species emerged, and heavier suffered population even gain a higher population density (Guo Cheng personal observation). Furthermore, it is also possible that other aspects of this species’ ecology, such as if the feeding range size of LDP individuals shows the same trend with their home ranges, and if their time budget, body condition may also change in response to human activity to improve survival as has been observed in other animals require further study. (Poudel et al., 2015b; Santini et al., 2019; Wright et al., 2010; Yang et al., 2019).

## CONFLICT OF INTEREST

The authors declare that they have no competing interests.

## AUTHOR CONTRIBUTION

**Shuailing Zhou:** Data curation (lead); Formal analysis (lead); Investigation (lead); Methodology (lead); Visualization (lead); Writing‐original draft (equal). **Ali Krzton:** Writing‐review & editing (equal). **Shuai Gao:** Data curation (supporting); Visualization (equal). **Cheng Guo:** Conceptualization (lead); Project administration (equal); Supervision (equal); Writing‐original draft (equal). **Zuofu Xiang:** Funding acquisition (lead); Project administration (equal); Resources (lead); Supervision (equal); Writing‐review & editing (equal).

## Supporting information

Fig S1

Fig S2

Fig S3

Fig S4

Fig S5

Fig S6

Table S1

## Data Availability

The datasets supporting this article are provided as Table S1.
