# Supplementary figures and images for: Effects of human activity on the habitat utilization of Himalayan marmot (Marmota himalayana) in Zoige wetland
Source: Ecol Evol. 2021 Jun 7;11(13):8957–68. doi: 10.1002/ece3.7733 (PMC8258216; doi:10.1002/ece3.7733)

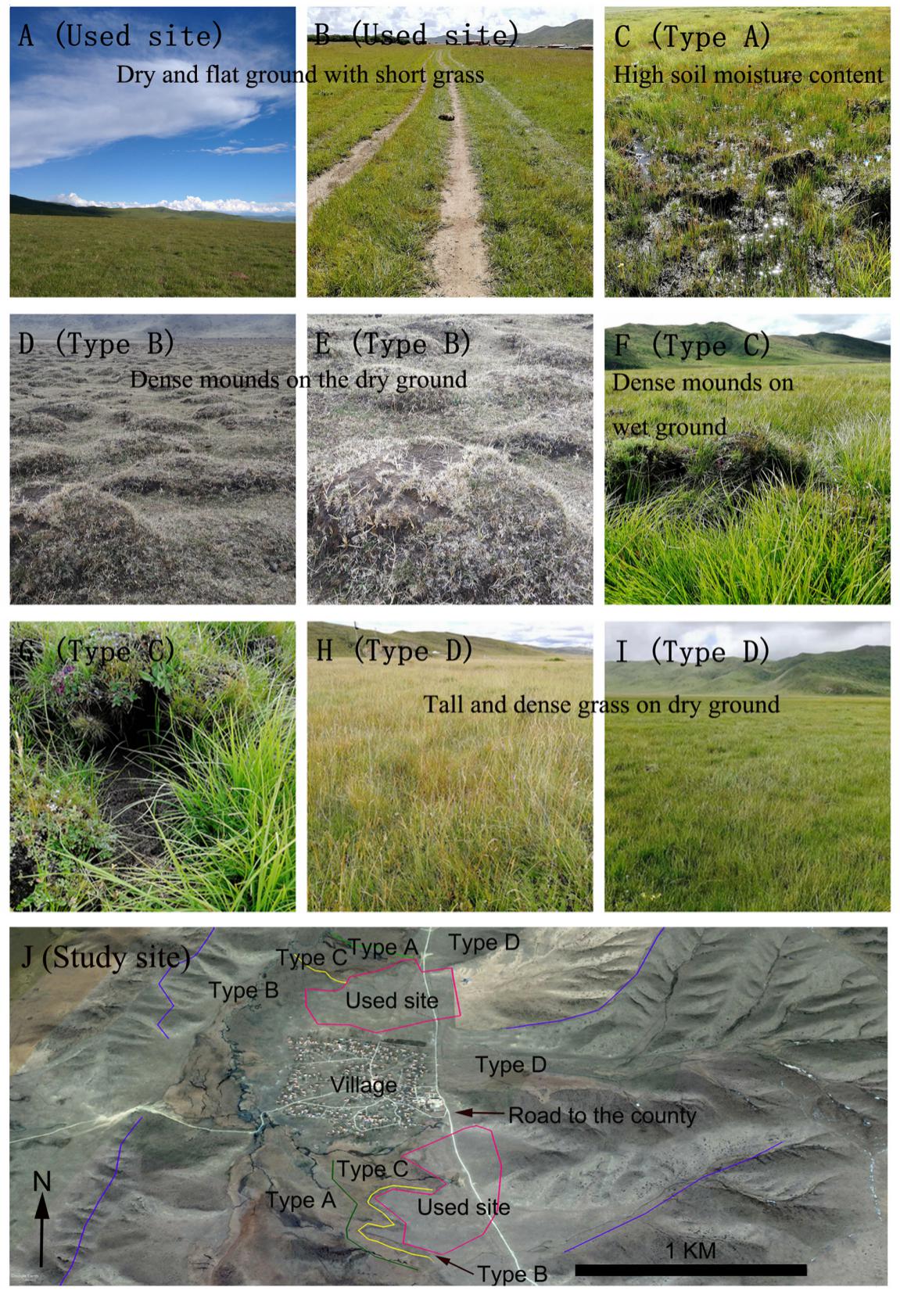


**FIGURE S1** Habitat type and microhabitat selection of Himalayan marmot in our study site.

Supplement: Supplementary file 1 — Fig S1 [file ECE3-11-8957-s007.docx]
