# Supplementary material for: Effects of human activity on the habitat utilization of Himalayan marmot (Marmota himalayana) in Zoige wetland
Source: Ecol Evol. 2021 Jun 7;11(13):8957–68. doi: 10.1002/ece3.7733 (PMC8258216; doi:10.1002/ece3.7733)
Supplement: Supplementary file 2 — Fig S2 [file ECE3-11-8957-s004.docx]

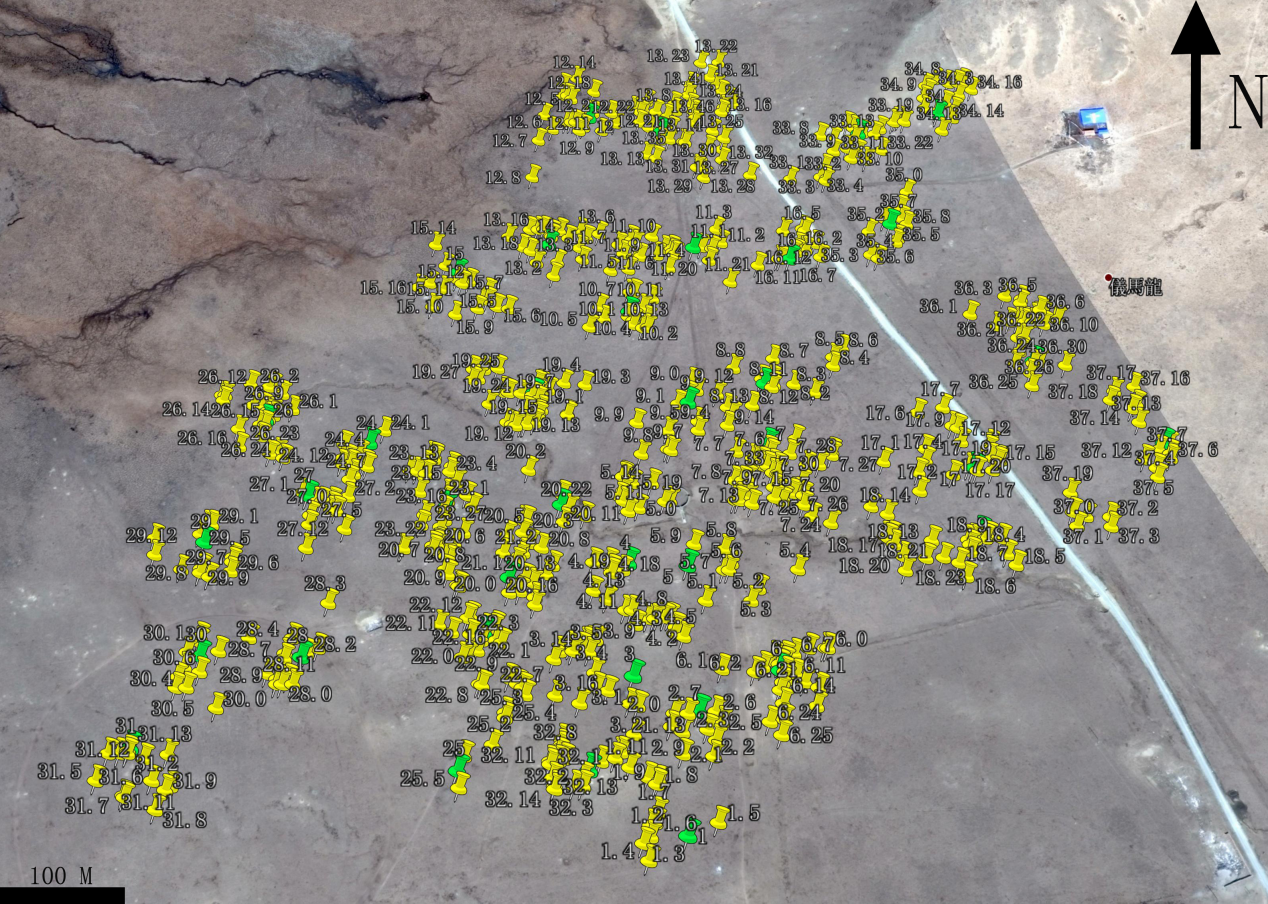


**FIGURE S2** location of all burrows in high disturbed habitat, reproductive burrows are showed in green pushpins and the temporary burrows are showed in yellow pushpins. Numbers next to the pushpins are I.D. of burrows (i.e. 1 represents the reproductive burrow of HDH1 breeding pair, and 1.5 represents the fifth temporary burrow we found during the field study).
