# Supplementary material for: Effects of human activity on the habitat utilization of Himalayan marmot (Marmota himalayana) in Zoige wetland
Source: Ecol Evol. 2021 Jun 7;11(13):8957–68. doi: 10.1002/ece3.7733 (PMC8258216; doi:10.1002/ece3.7733)
Supplement: Supplementary file 3 — Fig S3 [file ECE3-11-8957-s002.docx]

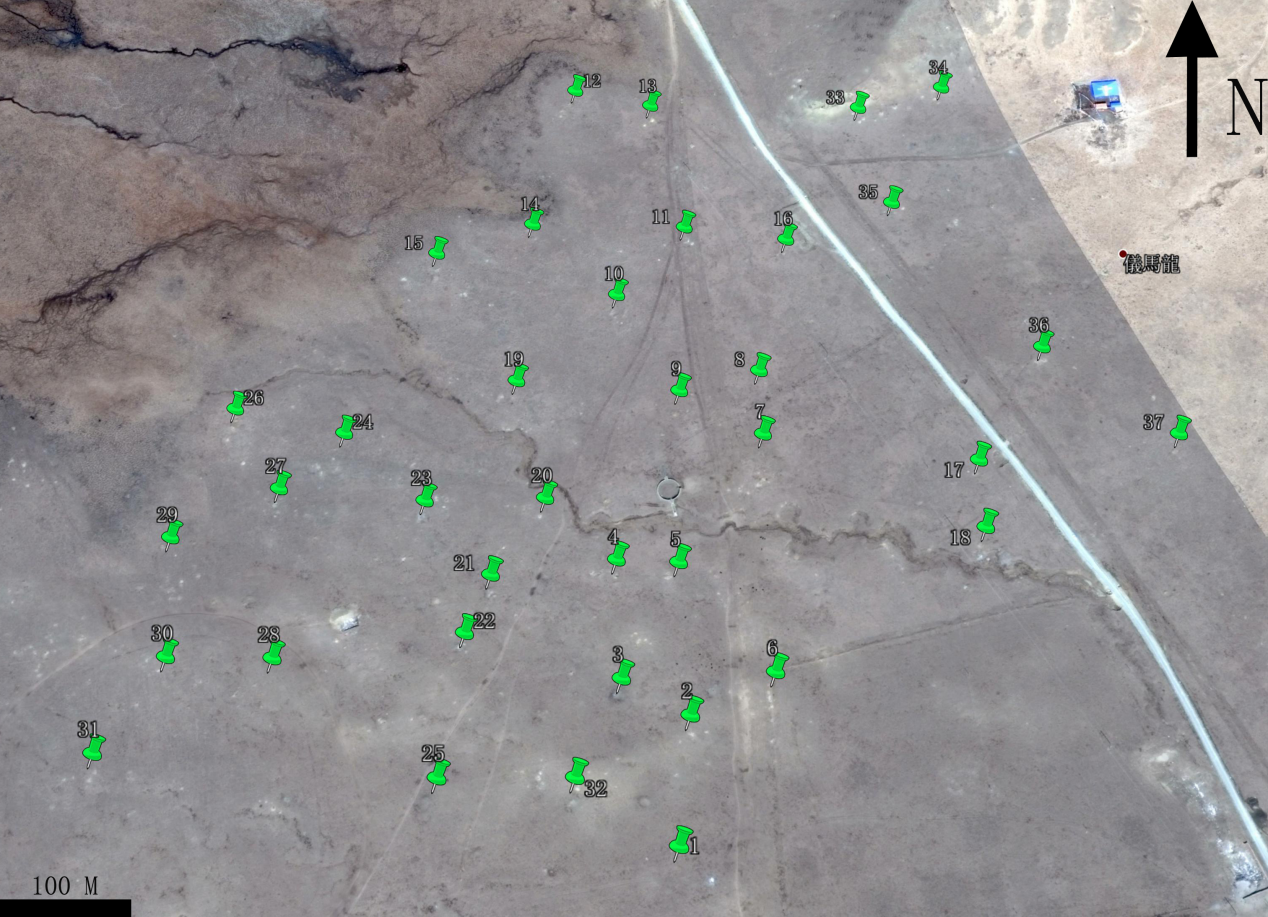


**FIGURE S3** Location of reproductive burrows in high disturbed habitat. Numbers next to the pushpins are I.D. of burrows (i.e. 1 represents the reproductive burrow of HDH1 breeding pair).
