# Supplementary material for: Effects of human activity on the habitat utilization of Himalayan marmot (Marmota himalayana) in Zoige wetland
Source: Ecol Evol. 2021 Jun 7;11(13):8957–68. doi: 10.1002/ece3.7733 (PMC8258216; doi:10.1002/ece3.7733)
Supplement: Supplementary file 4 — Fig S4 [file ECE3-11-8957-s005.docx]

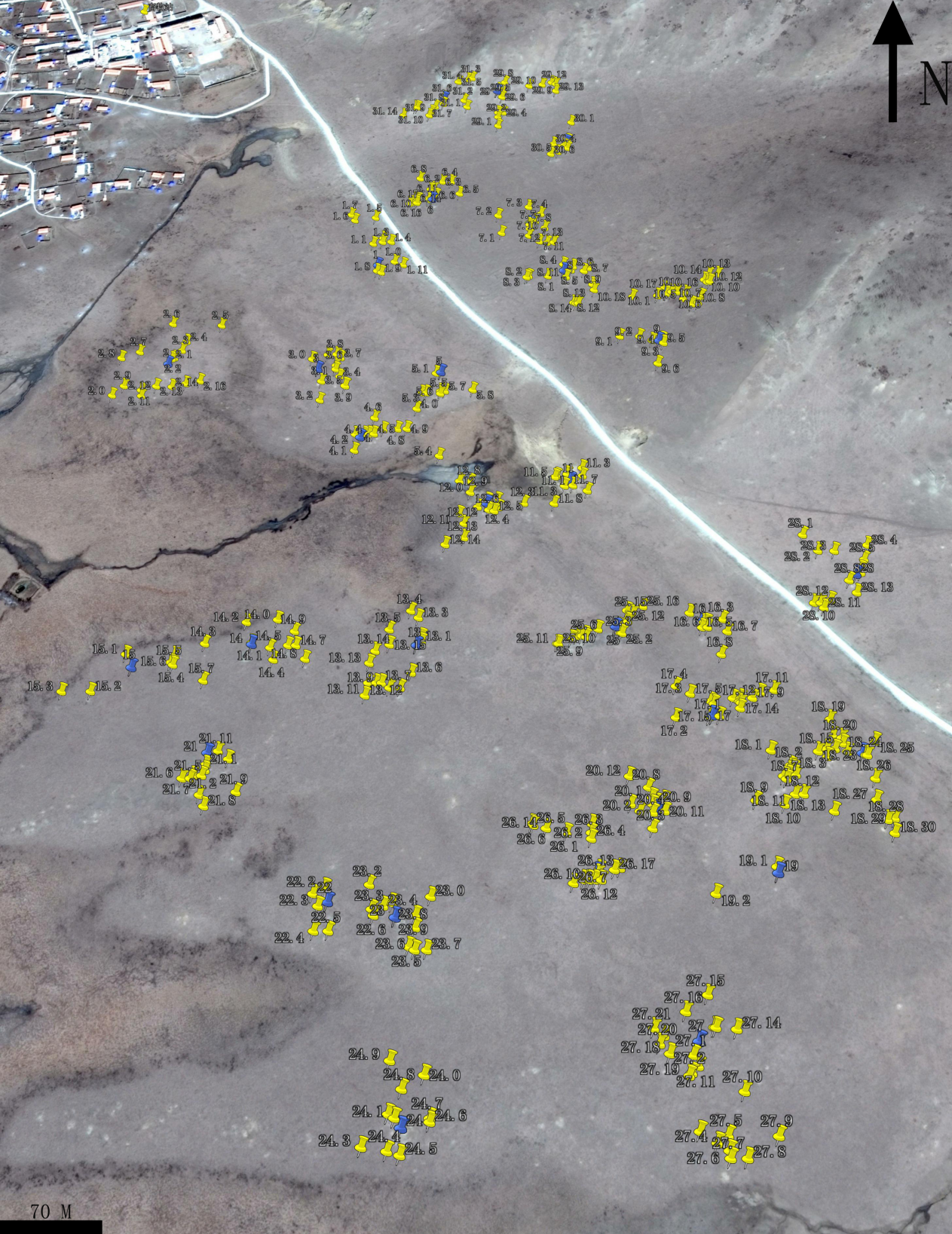


**FIGURE S4** Location of all burrows in low disturbed habitat, reproductive burrows are showed in blue pushpins and the temporary burrows are showed in yellow pushpins. Numbers next to the pushpins are I.D. of burrows (i.e. 24 represents the reproductive burrow of HDH24 breeding pair, and 24.5 represents the fifth temporary burrow we found during the field study).
