# Supplementary material for: Effects of human activity on the habitat utilization of Himalayan marmot (Marmota himalayana) in Zoige wetland
Source: Ecol Evol. 2021 Jun 7;11(13):8957–68. doi: 10.1002/ece3.7733 (PMC8258216; doi:10.1002/ece3.7733)
Supplement: Supplementary file 5 — Fig S5 [file ECE3-11-8957-s003.docx]

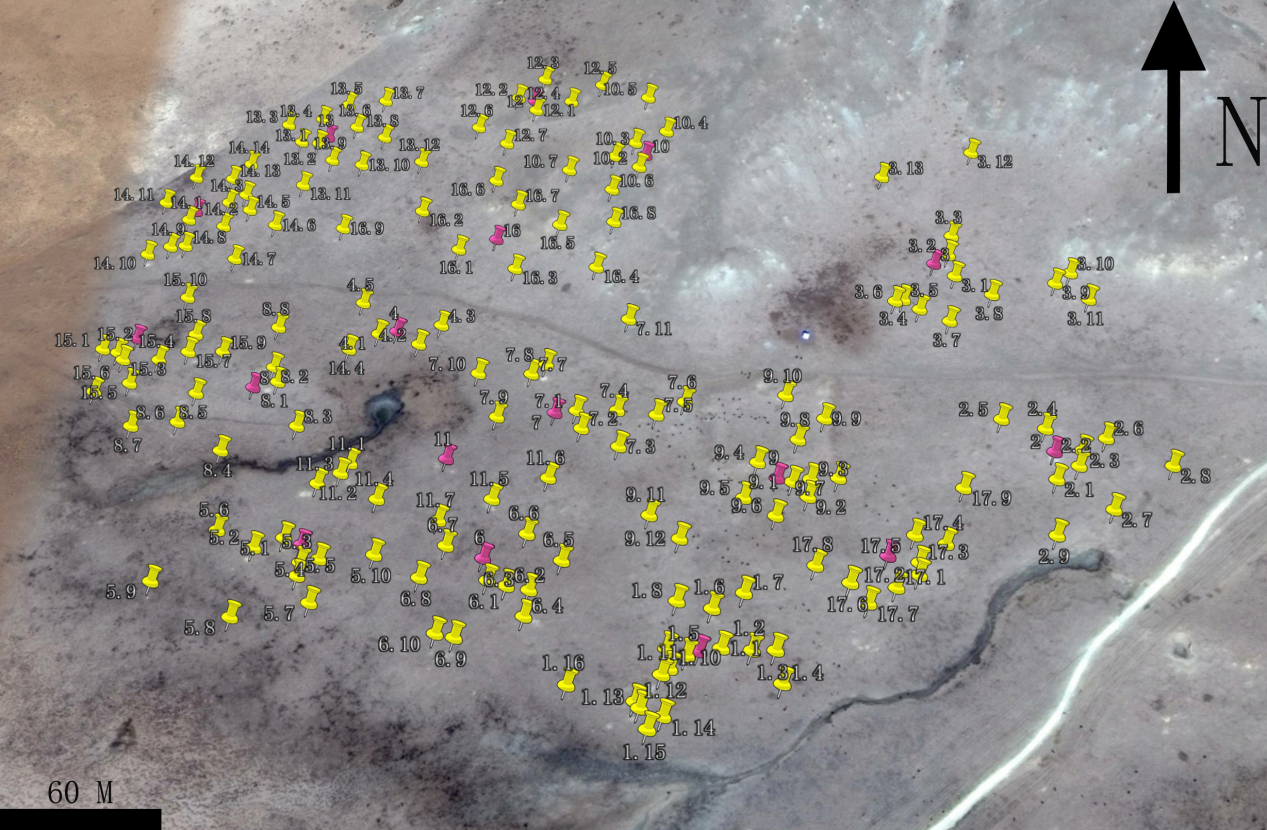


**FIGURE S5** Location of all burrows in natural habitat, reproductive burrows are showed in pink pushpins and the temporary burrows are showed in yellow pushpins. Numbers next to the pushpins are I.D. of burrows (i.e. 17 represents the reproductive burrow of HDH17 breeding pair, and 17.7 represents the seventh temporary burrow we found during the field study).
