# Supplementary material for: Effects of human activity on the habitat utilization of Himalayan marmot (Marmota himalayana) in Zoige wetland
Source: Ecol Evol. 2021 Jun 7;11(13):8957–68. doi: 10.1002/ece3.7733 (PMC8258216; doi:10.1002/ece3.7733)
Supplement: Supplementary file 6 — Fig S6 [file ECE3-11-8957-s001.docx]

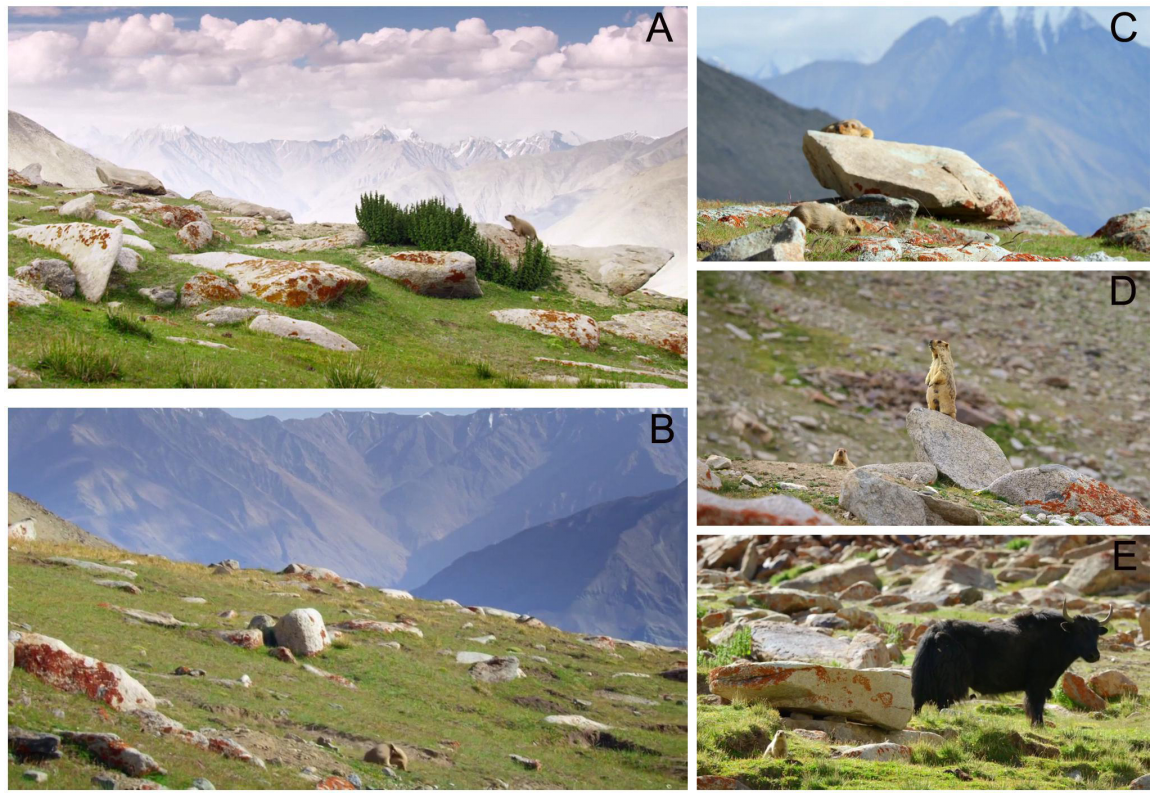
**FIGURE S6** Life of Himalayan marmot in an alpine meadow ecosystem. (A, B) habitat selected by the Himalayan marmot in the alpine meadow; (C) an individual is basking on a big stone; (D) an individual is watching on a big stone; (E) an individual is siting on the entrance of its burrow next to a big stone. All pictures were captured from a BBC documentary (Davina McCall, Mountains: Life at the extreme E2 Himalaya, 2017).
